# A Rare Cause of Infection Following Total Knee Arthroplasty: Abiotrophia defectiva Linked to Recurrent Urinary Tract Infections

**DOI:** 10.7759/cureus.80460

**Published:** 2025-03-12

**Authors:** Anas Nooh, Bashar Reda, Raed M Sharaf, Mohammed Alsubaie, Faisal F Alzahrani

**Affiliations:** 1 Orthopedic Surgery, Faculty of Medicine, King Abdulaziz University, Jeddah, SAU; 2 General Practice, Faculty of Medicine, King Abdulaziz University, Jeddah, SAU; 3 Orthopedic Surgery, Security Forces Hospital, Makkah, SAU

**Keywords:** abiotrophia defectiva, case report, prosthetic joint infection, total knee arthroplasty, urinary tract infection

## Abstract

*Abiotrophia defectiva* (*A. defectiva*) is a rare cause of prosthetic joint infection (PJI), and its role in surgical site infections remains unclear. A 66-year-old female developed an *A. defectiva *infection two years following primary right total knee arthroplasty (TKA). The patient presented with fever, knee pain, and urinary tract infection (UTI). She had a history of recurrent UTIs, likely contributing to the hematogenous spread of the pathogen. Knee aspiration was initially negative; however, subsequent cultures detected* A. defectiva*. The PJI was managed with surgical debridement, removal of prosthetic components, and a dynamic antibiotic spacer. We highlight the challenges associated with *A. defectiva *PJIs. The pathogen's fastidiousness complicates identification, and its biofilm formation on prosthetic materials makes eradication difficult. Despite its rarity, *A. defectiva *should be considered as a possible cause of PJIs, particularly in patients with recurrent UTIs. Early recognition and aggressive management are crucial.

## Introduction

*Abiotrophia defectiva (A. defectiva)*, a rare and fastidious organism, belongs to the nutritionally variant streptococci (NVS) group. Originally classified as Streptococcus, this bacterium was reclassified considering its unique growth requirements and pathogenic profile [1]. *A. defectiva* typically resides in the oropharynx and gastrointestinal and urogenital tracts as part of the normal human microbiota [2]. Yet, *A. defectiva* is a notable pathogen when introduced into sterile environments, where it can cause severe infection, including bacteremia and infective endocarditis [3-5]. These infections are often associated with high morbidity and mortality, particularly due to the propensity for endocardial damage and relative resistance to standard antimicrobial therapies [1].

Although the clinical relevance of *A. defectiva* has been increasingly recognized, its involvement in postsurgical infections remains underreported. *A. defectiva* is commonly linked to endocarditis, especially in patients with underlying valvular heart disease or prosthetic valve implantation [4,6]. However, musculoskeletal infections, particularly of prosthetic joints, are exceedingly rare. This rarity poses significant diagnosis and management challenges, as clinical manifestations can be nonspecific and the organism is difficult to culture using standard methods [6-8].

Periprosthetic joint infections (PJIs) are among the most serious complications of total knee arthroplasty (TKA), often necessitating complex revision surgeries and prolonged antibiotic therapy, occasionally causing permanent disability [9-11]. Most PJIs are caused by common pathogens; conversely, infections caused by organisms such as *A. defectiva* are rarely documented, making this case particularly noteworthy [12-16].

We present a rare case of an *A. defectiva* PJI following primary right TKA. We aimed to contribute to the growing understanding of *A. defectiva* as an emerging pathogen in the context of orthopedic infections and share our experience in managing this complex clinical scenario.

## Case presentation

A 66-year-old female presented with a significant medical history of hypertension, hypothyroidism, and osteoporosis. She underwent left and right total knee replacements (TKRs) to address severe osteoarthritis, performed in August 2022 and March 2023, respectively. Initial recovery was uneventful until the patient developed persistent right knee pain, erythema, and swelling in June 2024. Unfortunately, the patient’s infection was only partially treated with amoxicillin-clavulanate prescribed by a general physician without laboratory workup or joint aspiration. The patient sought further evaluation at our orthopedic clinic after persistent pain and swelling. C-reactive protein (CRP) and erythrocyte sedimentation rate (ESR) are presented in Table 1. Joint aspiration culture was negative. Close observation was conducted and partial improvement was noted. However, a month after, symptoms recurred and CRP and ESR were 48.3 mg/L and 92 mm/h, respectively, while joint aspiration demonstrated frank pus (Table 2). Therefore, urgent surgical debridement and the first stage of planned right TKR revision surgery were performed. The patient reported frequent urinary tract infections (UTIs) over the past year, often characterized by urgency, dysuria, and occasional hematuria; not all episodes were treated with antibiotics. Joint aspiration revealed Gram-positive cocci in clusters and chains, later identified as *A. defectiva*. Bacterial workup revealed urinary *Escherichia coli (E. coli)*, while blood cultures were negative. Transesophageal echocardiography was performed, which was negative for endocarditis.

**Table 1:** Laboratory workup first evaluation. CRP: C-reactive protein; ESR: erythrocyte sedimentation rate

Test	Value	Normal range
CRP	8.59 mg/L	0-3 mg/L
ESR	97 mm/h	0-30 mm/h

**Table 2:** Laboratory workup second evaluation. CRP: C-reactive protein; ESR: erythrocyte sedimentation rate

Test	Value	Normal range
CRP	48.3 mg/L	0-3 mg/L
ESR	92 mm/h	0-30 mm/h

**Figure 1:**
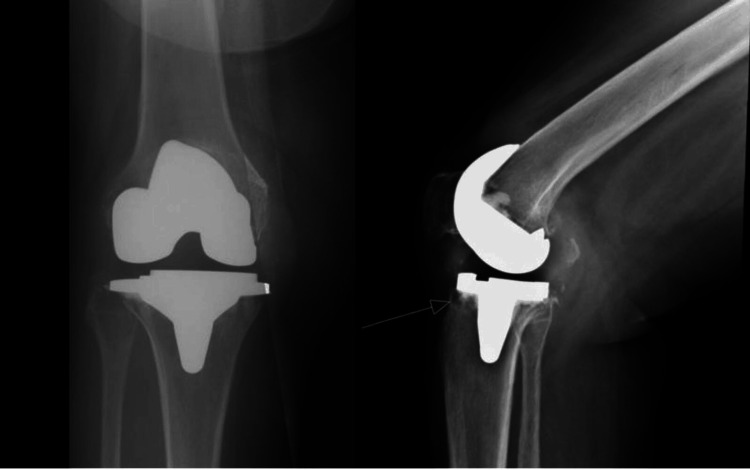
Preoperative lateral and anteroposterior views of the right knee joint.

**Figure 2:**
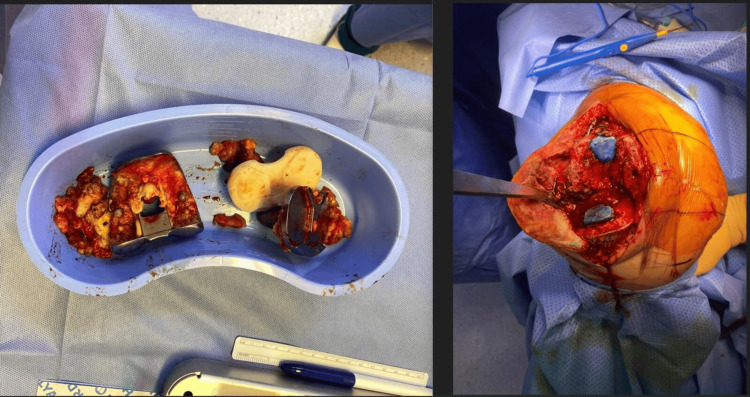
Removal of the femoral and tibial components and insertion of the antibiotic-loaded cement.

Figure 1 shows the preoperative radiographs of the right knee. After routine preoperative preparation, a median parapatellar arthrotomy was performed with a quadriceps snip under general anesthesia. The joint was opened, pus was noted, and multiple cultures were obtained from the joint space. The femoral and tibial components were extracted with minimal bone loss. Aggressive debridement of infected tissues was performed, and all residual cement was meticulously removed. A pocket of pus was discovered anterior to the fibular head, which was opened and thoroughly debrided. The knee was then subjected to an extensive washout with iodine, after which the wound was loosely closed, and regowning and redraping of the surgical site were performed to maintain sterility. Femoral and tibial cement dowels were prepared by mixing 3 g of vancomycin and 1 g of gentamycin with one pack of cement. These dowels were inserted in the femoral and tibial canals to provide intramedullary antibiotic elution (Figure 2).

**Figure 3:**
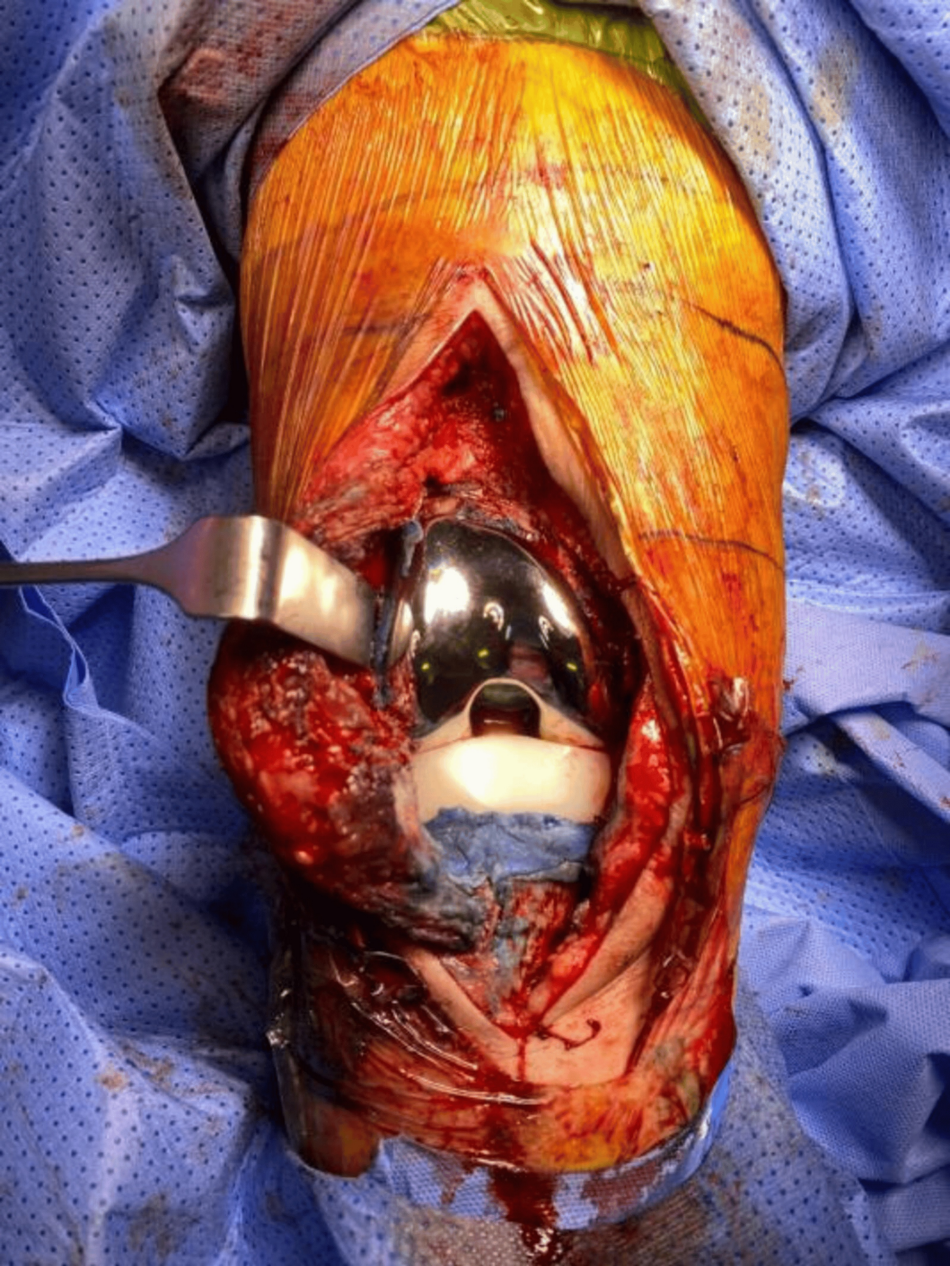
Intraoperative view of the right knee antibiotic knee spacer.

**Figure 4:**
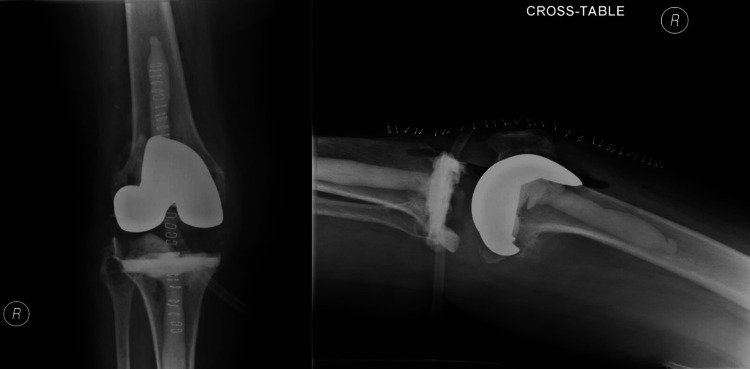
Postoperative cross-table and anteroposterior views of the right antibiotic knee spacer.

A posterior-stabilized femoral component was cemented in place, followed by the application of a cement layer to the tibial surface. Cement was prepared as described above. A trial of the polyethylene insert was performed to ensure proper alignment, range of motion, and stability. Once satisfied with the positioning, we cemented a posterior-stabilized polyethylene insert on the tibia (Figures 3, 4).

The arthrotomy and the subcutaneous tissues were closed in layers using monofilament sutures following standard procedures, and the skin was closed using surgical clips. An incisional vacuum-assisted closure therapy dressing was applied, and the patient was stable when transferred to the recovery room. The tourniquet was deflated after 120 min and hemostasis was confirmed with an estimated blood loss of 300 mL. The patient received intravenous antibiotics consisting of ceftriaxone 2 g and teicoplanin 850 mg for six weeks followed by oral antibiotics for another six weeks. The patient recovered uneventfully and regained her previous functional status immediately. The last CRP and ESR values are presented in Table 3. The patient is free of infection and due for second-stage revision in three months.

**Table 3:** Laboratory workup third evaluation. CRP: C-reactive protein; ESR: erythrocyte sedimentation rate

Test	Value	Normal range
CRP	23.3 mg/L	0-3 mg/L
ESR	91 mm/h	0-30 mm/h

## Discussion

*A. defectiva* infection during TKA is rare [12,14]. Although *A. defectiva* forms part of the normal human flora, its role as a pathogen in PJIs is unusual and poses unique diagnosis and management challenges. The pathogenesis of *A. defectiva* infection in this case likely stemmed from the patient's recurrent UTIs. Since *A. defectiva* is commonly found in the urogenital tract, it is plausible that the pathogen entered the bloodstream during one of these episodes, subsequently infiltrating the prosthetic joint [12,15,17,18]. The patient's history of chronic UTI suggested a possible breach in the mucosal barriers, facilitating bacterial translocation and hematogenous spread. Although the initial cultures from the knee aspirate were negative, the eventual identification of *A. defectiva* highlights its potential to cause delayed or indolent infections that may not present with classic signs of acute sepsis [12,14].

The literature on *A. defectiva* as a causative agent of TKA infections is sparse. Our study and literature review revealed a total of 10 cases of *A. defectiva* knee infection, eight of which occurred in prosthetic knees [12-16]. Most reported cases involve infections in other prosthetic devices or locations such as cardiac valves, where *A. defectiva* shows a propensity to cause biofilm-associated infections. Biofilms are difficult to eradicate and contribute to the chronicity and recurrence of PJIs. In TKAs, the development of a biofilm on prosthetic surfaces can shield *A. defectiva* from the host immune response and antibiotic therapies, making the infection particularly resilient to standard treatments [3,12,15].

The clinical course of our patient underscores the diagnostic and therapeutic challenges associated with *A. defectiva* infections. The initial presentation of fever, knee pain, and symptoms of UTI led to a differential diagnosis that included both urinary and joint infections. However, the negative culture from the initial knee aspiration and the presence of *E. coli* in the urine culture complicated the diagnostic process, as *E. coli* is a more common uropathogen and might have been presumed to be the primary cause of her symptoms. It was only upon repeated aspiration and culture that *A. defectiva* could be identified, guiding appropriate surgical and antimicrobial interventions [1,4,12].

The management of *A. defectiva* infections, particularly in TKAs, requires a multifaceted approach. Surgical intervention, as demonstrated in this case, is crucial for eradicating infection, especially considering the likelihood of biofilm formation [14,15]. The use of antibiotic-laden cement during revision surgery serves a dual purpose - providing joint mechanical stability and delivering high local concentrations of antibiotics directly to the infection site. In this case, vancomycin and gentamycin were incorporated into the cement, targeting both Gram-positive cocci, including *A. defectiva*, and other potential pathogens. This strategy is consistent with literature that supports the use of antibiotic-loaded cement for managing PJIs, particularly with resistant or uncommon organisms [13,15,16].

The challenges in diagnosing and treating *A. defectiva* infections extend to the laboratory. *A. defectiva* is a fastidious organism that requires specific nutritional supplements for growth in culture which may not be present in standard media. This may explain the initial negative culture results and underscores the importance of considering (NVS) in culture-negative PJIs, particularly with high clinical suspicion of infection [12,16].

This case highlights the need for increased awareness of rare pathogens, such as *A. defectiva*, in patients with recurrent UTIs who present with postoperative infection. It also emphasizes the potential link between distant infections and the development of PJIs. A multidisciplinary approach involving orthopedic surgeons, infectious disease specialists, and microbiologists is essential for optimizing patient outcomes. Early recognition, preoperative assessments, appropriate surgical intervention, postoperative monitoring, and tailored antimicrobial therapy are key to managing complex infections and preventing recurrence.

## Conclusions

This study emphasizes the clinical significance of *A. defectiva* as an uncommon but serious pathogen in TKA. The patient's history of recurrent UTIs likely contributed to the hematogenous spread of this organism. The challenges faced in diagnosing and treating *A. defectiva* infections highlight the importance of considering rare pathogens in the differential diagnosis of postoperative infections, particularly in patients with predisposing factors, such as recurrent UTIs. The successful management through revision surgery and the use of antibiotic-loaded cement underscores the importance of a comprehensive and aggressive approach to treating PJIs caused by uncommon organisms.
